# Predicting behavioral and brain markers of inhibitory control at preschool age from early measures of executive attention

**DOI:** 10.3389/fpsyg.2023.983361

**Published:** 2023-03-02

**Authors:** Ángela Conejero, Josué Rico-Picó, Sebastián Moyano, Ángela Hoyo, M. Rosario Rueda

**Affiliations:** ^1^Department of Developmental and Educational Psychology, University of Granada, Granada, Spain; ^2^Mind, Brain and Behaviour Research Centre (CIMCYC), University of Granada, Granada, Spain; ^3^Department of Experimental Psychology, University of Granada, Granada, Spain

**Keywords:** inhibitory control, infancy, toddlerhood, early childhood, longitudinal research, executive attention

## Abstract

**Background:**

Inhibitory control (IC) is the ability to prevent prepotent responses when inappropriate. Longitudinal research on IC development has mainly focused on early childhood and adolescence, while research on IC development in the first years of life is still scarce. To address this gap in the literature, we explored the association between executive attention (EA) and elementary forms of IC in infancy and toddlerhood, with individual differences in IC later at 5 years of age.

**Method:**

We conducted a five-wave longitudinal study in which children’s EA and IC (*n* = 96) were tested at the age of 9 and 16 months and 2, 3, and 5 years. Children performed various age-appropriate EA and IC tasks in each wave, measuring inhibition of attention, endogenous control of attention, inhibition of the response, and conflict inhibition. At 5 years of age, IC was measured with a Go/No-go task while recording event-related potentials. After correlation analyses, structural equation model analyses were performed to predict IC at 5 years of age from EA and early IC measures.

**Results:**

The results revealed that EA at 9 months predicted IC measures at 2 years of age. Likewise, measures of IC at 2 years predicted performance on the Go/No-go task at behavioral and neural levels. No direct association was found between EA at 9 months and IC at 5 years of age. We further observed that some EA and IC measures were not associated across time.

**Conclusion:**

As we expected, EA skills in infancy and toddlerhood were related to better performance of children on IC tasks, toghether with a more mature inhibition-related brain functioning. Altogether, the results indicate that IC in early childhood could be predicted from EA and IC at 9 months and 2 years of age and suggest that the early emergence of IC relies on the development of particular EA and basic IC skills. However, some discontinuities in the longitudinal development of IC are observed in the first 5 years of life. These findings provide further support for the hierarchical model of IC development.

## 1. Introduction

Entering formal education at preschool age poses an important challenge for children. Preschoolers experience increased learning demands and have to adapt to a structured environment in which they are required to behave according to rules. To succeed in this endeavor, children must develop what has been called inhibitory control (IC), which refers to the ability to suppress a prepotent response or irrelevant information to meet some goal or context demands (Diamond, 2013). It is one of the three main components of executive functions according to the Miyake and Friedman model (Miyake et al., 2000; Friedman and Miyake, 2017). There is some evidence indicating that the first 5 years of life might be crucial for IC development (Garon et al., 2008). During this time, IC skills undergo rapid changes, showing a steadier pace throughout childhood and adolescence (Klenberg et al., 2001; Simpson and Riggs, 2006; Ordaz et al., 2013). These findings suggest that individual differences in IC become stable to some extent from early childhood. In support of this suggestion, individual differences in IC skills of children around the fifth birthday can predict important developmental outcomes, such as academic success or later social adjustment in adulthood (McClelland and Cameron, 2011; Mof et al., 2011). However, the development of IC up until the age of 5 years is still underexplored. The literature on IC development focuses more on the period from childhood to adolescence. For many years, research has neglected IC at younger ages. There is still limited published data on how individual differences in IC initially develop within the first years of life (Hendry et al., 2016). One of the main reasons for this gap in the field is that IC is better measurable in older children compared to infants and toddlers. In general terms, pre-verbal children are limited in their language and motor skills, decreasing the reliability with which they can perform most of the classic IC paradigms (Conejero and Rueda, 2017).

One of the cognitive tasks more widely used for measuring IC in children and adults is the Go/No-go task. This task has two main strengths: It allows for measuring different aspects of IC and can be easily adapted for measuring brain activity associated with the target processing with electroencephalography (EEG). The task involves pressing a button as soon as the Go stimulus is perceived, withholding the response when a No-go stimulus appears. In order to challenge IC, the task generates a tendency to respond by including a greater proportion of Go trials (approximately 75%). The performance of children in this task improves with age (Jonkman, 2006; Wiebe et al., 2012) so that they commit fewer errors in the No-go condition and increase their differential response to the Go and No-go conditions (to which d’ from signal detection theory is calculated) and a lower proportion of omissions (missing responses in Go trials). Whereas the first two measures are used as indicators of IC deployment, omissions are viewed as a sign of a decline in sustained attention during the task (Lewis et al., 2017a). Regarding neural markers of IC in the Go/No-go task, EEG studies have identified two main event-related potentials (ERPs): the N2 and P3 components. The N2 is frontocentral negativity peaking approximately 200–300 ms after stimulus onset. Conversely, the P3 component is a positive component more prominently observed in parietal sites between 300 and 600 ms after stimulus onset. Increased amplitudes of N2 and P3 have been reported for No-go trials in both children and adults and are thought to reflect the recruitment of IC (Jonkman et al., 2003; Gajewski and Falkenstein, 2013). In fact, larger amplitudes of the No-go N2 and P3 components are considered to reflect a more mature IC in young children as it is also associated with better performance in the task (Cragg et al., 2009; Willner et al., 2015; Abdul Rahman et al., 2017; Swingler et al., 2018). Overall, this task can provide a very good picture of individual differences in IC processes in early childhood at both behavioral and neural levels.

Although IC can be relatively well characterized in children, less is known about how these early differences in IC develop during the first years of life. The bulk of the research on the early development of IC has investigated the changes in this cognitive ability in children from 2 years of age onward. One exception is the recent study by Neale et al. (2018) that predicted IC of 2-year-olds from their ability in infancy to inhibit grasping a spoon from the nearest reaching side when it did not match with the handle location. Otherwise, most of the longitudinal studies on IC investigated age-related changes within the 2 and 5 years of age in various IC tasks tapping different IC skills, such as inhibiting a prepotent response, the ability to delay gratification, or the efficiency in dealing with cognitive conflict. This is the case of Kochanska et al. (1997, 2000), who were the first researchers to develop a battery of behavioral tasks measuring IC in toddlers. Tasks in this battery assessed the ability to inhibit behavior in the delay of gratification tasks and to suppress an ongoing activity. These authors informed a linear improvement in IC across the ages 22–33 months (Kochanska et al., 2000) and 33–66 months (Kochanska et al., 1997), with IC at younger ages predicting IC as children grow older. Later longitudinal research on the development of IC followed a similar approach, measuring IC across time with a set of age-appropriate tasks at each time point in order to expand the age range in which children could be longitudinally tested. However, studies have yielded inconsistent results. This is evidenced in the study by Kloo and Sodian (2017), who observed that IC at 30 months, but not at younger ages, was associated with the IC ability of 5-year-olds. Moreover, Joyce et al. (2016) found that although IC in delay tasks was correlated over time between the 2nd and 4th year of age, the performance of children in conflict tasks was unrelated across ages suggesting that during toddlerhood, individual differences in delay, the ability can be more stable compared to other aspects of IC such as conflict inhibition. Conversely, a lack of stability in the individual differences in the ability to delay has also been reported between 2 and 3 years of age (Gagne and Saudino, 2016), as well as a stable enhancement in conflict inhibition between 2 and 4 years of age (Hughes and Ensor, 2005). Overall, results from these studies may indicate that IC in early childhood can be predicted from toddlerhood. However, it should be expected that a discontinued trajectory in the development of IC during toddlerhood may depend on the IC ability being examined.

A few studies have attempted to address the longitudinal changes in IC from earlier ages, some of them starting in infancy or early toddlerhood. However, data from these studies suggest that the developmental trajectory of IC skills is more unstable over this period. For example, Gagne and Saudino (2016) observed that children’s score in the IC scale of the Lab-TAB (which rates children’s temperament behavior and emotional reactions in standard lab situations) was not longitudinally associated between 12 and 36 months of age. Likewise, Miller and Marcovitch (2015) observed that the performance of children in the A-not-B task was uncorrelated across ages between 14 and 18 months of age. The A-not-B task requires children to inhibit looking for an appealing object (e.g., a toy) in the location where this object was initially hidden (A) to successfully retrieve such object in a new different location (B). A poor performance in the A-not-B task at 14 months (i.e., perseverating more in searching for the object in location A after switching the object to location B) was not necessarily maintained at the age of 16 months. A similar pattern of results was found by Hendry et al. (2021) between 10 and 16 months of age. Authors found that infants’ IC in the A-not-B task was unrelated to their performance 6 months later in the Early Childhood Inhibitory Control Touch Screen Task, which measures toddlers’ ability to refrain from touching the location where the target appears with a higher probability (prepotent response) in the occasions that target appeared in the opposite side.

The apparent lack of longitudinal stability of early measures of IC could be reflecting the heterogeneity in the development of IC processes [for example, see ref. (Hendry et al., 2021) that recently distinguished between competing inhibition, directed global inhibition, and behavioral inhibition]. Some authors argue that the development of IC follows a hierarchical structure (Garon et al., 2008; Hendry et al., 2016). According to this idea, IC development is built from more basic IC-related skills that emerge at younger ages. In line with this theoretical framework, some studies tried identifying possible cognitive precursors of IC in infancy and toddlerhood. Among others, emerging attention skills have been consistently proposed to form the foundation for IC. Some prior research evidenced that infants’ general ability to focus and sustain attention in free-play situations is associated with IC abilities at about the 2nd year of age (Kochanska et al., 2000; Johansson et al., 2015). Regarding specific attention skills, for many years, research has drawn attention to the link between executive attention (EA) development and IC individual differences in toddlerhood and early childhood (Rueda, 2014). According to the attention network model (Posner and Petersen, 1990; Petersen and Posner, 2012), Executive Attention is one of the three main functions of attention. EA refers to the control of attention and comprises the inhibition of distractors, detecting conflicting information or attention shifting. This construct partially overlaps with IC (Diamond, 2013; Friedman and Miyake, 2017), with the caveat that it seems to develop earlier than IC and can be easily measured from infancy: first signs of the control of attention become apparent from the 6th month of life (Courage et al., 2006).

Despite the literature generally agreeing that EA skills underlie the development of IC (Rothbart and Rosario, 2005; Rothbart et al., 2011; Wass, 2015), few studies to date have empirically tested this theoretical assumption with experimental tasks. Holmboe et al. (2008) found that 9-month-olds who showed better EA in the so-called Freeze Frame (i.e., by selectively suppressing their attention to peripheral stimuli as a function of the target interest) outperformed in a classic IC task (a Spatial Conflict task) at 2 years of age. As reported by Holmboe et al. (2018), in a more recent study investigating the longitudinal associations between EA and IC from 4 to 9 months of age, the direct link between EA and IC is not observed until 9 months, even though individual differences in EA remained stable from 6 to 9 months of age. This may indicate that late infancy would be a turning point in the development of EA and for the transition between EA and the emergence of IC skills. In addition, toddlers’ EA skills are also related to the later development of IC. The greater ability of 2-year-olds to anticipate the location of a target in a sequence was associated with better IC in a Spatial Conflict task (Rothbart et al., 2003), and the performance in a visual selection task at 30 months predicted children’s ability to delay gratification at 3 years of age (Veer et al., 2017). In fact, structures of the prefrontal cortex undergo a rapid development during the second half of the first year (Dean et al., 2014; Li et al., 2015), with a greater engagement of prefrontal brain structures in activities that require the control of attention between 14 and 16 months of age (Weibley et al., 2021). Some authors claimed that during this period, frontal brain regions come more prominently into play in the control of attention, taking over subcortical and parietal brain structures (Alcauter et al., 2014; Fiske and Holmboe, 2019). In light of this, 9 months of age could be settled as a good starting point to explore how EA skills lay the ground for IC development over the first years of life.

Altogether, existing evidence provides important insights into the development of IC during the first 5 years of life, while highlighting existing uncertainties around this matter. The current study aims to track back to infancy and toddlerhood the roots of the individual differences in IC observed by the age of 5 years. The current study intends to fill a gap in the current literature regarding the development of IC in the first years of life. Although a body of research exists exploring the changes in the IC capacity of children at different ages, few studies have addressed this issue longitudinally from early development. Some of the existing longitudinal studies focused on the first 2 years of life, whereas others start from 2 years of age, with none of them covering the full period between infancy to the 5th year of life. There is also a dearth of research about the involvement of basic IC-related skills that develop earlier, such as EA, in the emergence of IC. The current study explores potential precursors of IC at 5 years of age, such as EA and emergent IC skills in infancy and toddlerhood. For this purpose, we selected a set of age-appropriate EA and IC tasks to explore the longitudinal associations among measures in a five-wave longitudinal study. Tasks were selected according to their potential sensitivity to detect individual differences in EA or IC at each age (Carlson, 2005; Garon et al., 2008; Best and Miller, 2010). At 9 and 16 months of age, two different EA, eye-tracking tasks were administered as follows: A shifting task developed by Kovács and Mehler (2009) in which infants had to inhibit anticipatory looks toward a previously rewarded location that is no longer relevant at 9 months of age; a visual sequence learning task (Sheese et al., 2008) in which correct anticipatory looks to the target location in a sequence have been previously demonstrated to be linked to IC at 16 months of age. At 27 months of age, IC was assessed with the Reverse Categorization Task (Hongwanishkul et al., 2005), which measured children’s ability to inhibit the initial sorting rule once it is reversed (with a similar structure to the shifting task administered at 16 months of age); a Spatial Conflict task (Gerardi-Caulton, 2000) at 36 months of age, in which children have to override the tendency to touch the side where the target appears when the correct matching response locates in the contralateral position. In addition, children performed two different delay tasks at 27 and 36 months of age: the Snack Delay Task and the Delay of Gratification Task, respectively. Finally, a child-friendly version of the classic Go/No-go task (Casey et al., 1997) was administered by the age of 5 years as an outcome measure of IC. In this task, participants need to suppress a previously automatized response. In addition, we aimed to extend our knowledge about the individual differences in IC by also examining neural markers of IC. For this purpose, we registered EEG while children performed the Go/No-go task at 5 years of age. We expected that EA and IC measures would positively correlate across infancy and toddlerhood. However, considering prior studies, discontinuities in the association between EA and IC measures over time could also be expected. Furthermore, we anticipated that both behavioral and neural indicators of IC related to the performance of children in the Go/No-go at 5 years of age would be associated with EA and IC in infancy and toddlerhood. Consequently, children that exhibited better EA skills at 9 or 16 months of age and a more efficient IC at 2 or 3 years of age would (Diamond, 2013) outperform in the Go/No-go task, which would be translated into a lower proportion of omissions in go trials, diminished commission errors in No-go trials and increased sensitivity (d’) and (Friedman and Miyake, 2017) present enhanced N2 and P3 in the No-go condition. Finally, we tested the hypothesis that IC development follows a hierarchical structure by building a statistical model that accounted for individual differences in IC at 5 years of age from specific EA and IC measures at younger ages.

## 2. Methods

### 2.1. Participants

A total number of 98 infants (46 females) initially participated in the study at 9 months of age. Families with babies below 9 months of age were reached through advertisements in nurseries, local press and media, social networks, and the University of Granada bulletin board on their web page. All children included in the study were born at full term (>37 weeks of gestational age) and had no history of neurodevelopmental disorders or any other clinical psychological condition. Children participated in the study at five different time points: at 9 months of age (range: 9–12 months; mean = 10.7, SD = 1.55), at 16 months of age (range: 16–18 months; mean = 16.77, SD = 0.60), at 2 years of age (range: 25–26 months; mean = 26.63, SD = 0.88), at 3 years of age (range: 36–38 months; mean = 37.72, SD = 2.42), at and 5 years of age (range: 60–64 months, mean = 62.5, SD = 1.79). From the initial sample at 9 months, 88 children participated at the age of 16 months, 61 at the age of 2 years, 57 at the age of 3 years, and 52 at the age of 5 years. Retention rate across testing sessions was approximately 69% from the first to the second testing session, 87% from the second to the third testing session, 93% from the third to the fourth testing session, and 91% from the fourth to the fifth testing session. The number of children who provided enough usable data at each time point for every experimental task administered is provided in Table 1. The sample size for the different tasks ranged from 59 to 42. Families received a 10 € token for educative toys in each of the experimental sessions to compensate for their participation in the study.

**Table 1:** Descriptive data for all measured variables at the different waves of the study.

		Valid *n*	Min	Max	Mean	SD
9 months	Shifting task (% Perseverations)	59	0	100	52.37	32
16 months	Visual Sequence learning task (% correct anticipations)	51	0	75.86	29.68	2.78
2 years	Reverse categorization task (*z* score)	42	−3.16	1.44	0.00	1
	Snack Delay task (*z* score)	51	−3.21	0.66	0.00	1
3 years	Spatial conflict task (Conflict score, RT)	51	−1106.25	1036.75	57.93	419.93
	Spatial conflict task (Conflict score, ACC)	51	−0.33.50	0.33.50	0.10	14.22
	Delay of gratification task (*z* score)	53	−1.92	1.41	0.00	1.00
5 years	Go/No-go (% Omissions)	44	0.00	27.50	6.36	6.03
	Go/No-go (% No-go errors)	44	2.30	92.50	34.60	19.17
	Go/No-go (d’)	44	0.37	5.25	2.44	1.18
	Peak N2 No-go (μV)	43	−11.40	6.61	−3.29	3.83
	Difference peak N2 (μV)	43	−12.64	11.90	1.45	4.32
	Peak latency N2 Go (ms)	43	300	400	340.56	28.72
	Peak latency N2 No-go (ms)	43	300	400	335.63	28.31
	Peak P3 No-go (μV)	43	−11.78	6.61	3.25	4.70
	Difference peak P3 (μV)	43	−4.85	26.17	5.95	6.12
	Peak latency P3 Go (ms)	43	400	800	548.65	127.83
	Peak latency P3 No-go (ms)	43	400	744	548.19	69.53

### 2.2. Procedure

#### 2.2.1. General procedure

Once the caregivers agreed to participate, they were contacted to set a date for their first visit to the lab when the children turned 9 months of age. For follow-up sessions, the experimenter contacted families to schedule an appointment at their convenience as soon as children grew up to the established ages for each wave. Testing was carried out in the laboratory. At the beginning of each testing session, caregivers were told about the structure of the session and the characteristics of the tasks. They were also instructed not to interfere with children’s behavior and to remain silent during experimental tasks. After that, they were asked to sign informed consent. The testing session started following 5 min of warming up in which children were familiarized with the testing room and experimenter.

Children performed a set of different age-appropriate tasks measuring EA or IC-related skills at each session. Additional tasks not relevant to the purpose of this study and not reported in this article were also administered. A complete experimental session took approximately 45 min to 1 h. Short breaks were made between tasks or blocks of trials to avoid children’s fatigue. Additional breaks were taken at any moment when needed at younger ages (e.g., caregiver demanded to stop feeding the child or if signs of tiredness were observed in the child). All the procedures used in this research were approved by the University of Granada Ethics Committee and have been performed in accordance with the standards contained in the Declaration of Helsinki.

#### 2.2.2. Apparatus, materials, and measures

##### 2.2.2.1. Eye-tracking measures

Eye-tracking measures were administered at the age of 9 and 16 months. Children were seated on the caregiver’s lap in front of the screen in a deemed lightroom. Stimuli were presented in SMI Experiment Centre 3.2 software (SensoMotorics Instruments, Teltow, Germany), whereas infants’ looking behavior was recorded with corneal-reflection eye-tracker RED 250 by SensoMotorics Instruments (SMI) with iView X Hi-Speed (SensoMotorics Instruments, Teltow, Germany) system (temporal resolution = 250 Hz; spatial resolution = 0.03°). Stimuli were displayed on 1,024 × 768 pixels, 19-inch monitor (60 Hz). The experimenter stayed in a contiguous room controlling the experiment presentation while watching children’s behavior and gaze data registration. A 5-point child-friendly calibration (colorful looming points with sound located in the corners and center of the screen) was administered before starting each of the tasks. Saccades and fixations were computed according to the following parameters: peak velocity threshold = 40°/s; minimum fixation duration = 50 ms. We computed the proportion of looks to any of the defined areas of interest (AOIs) for each task during the anticipatory period. Anticipatory looks that occurred in the first 200 ms after the onset of the peripheral target were excluded, given that they are considered spurious more than a result of a real expectation (Canfield and Haith, 1991). Only trials with direct looks at one of the AOIs were included in subsequent analyses.

A shifting eye-tracking task was administered at the age of 9 months. The task was adapted from Kovács and Mehler (2009). Children have presented with two white boxes on the left and right positions within a black background on a screen (size: 18° × 18°, 15° eccentricity; AOIs size: 21° × 19°). Once children fixated on an attention-getter (an animated start with music centrally located), the trial is automatically initiated. After a 1 second delay (anticipatory period), an animated cartoon coupled with funny sound effects appeared in one of the boxes for 2 s. Stimuli appeared in the same location for nine consecutive trials in the first block, changing to the opposite side for another nine trials in the following second block (total number of trials = 18). The initial location of the cartoon (left or right) was counterbalanced across participants. Only children who completed at least 50% of total trials and had reliable eye-tracking data according to calibration information (*n* = 59) were included in the final analyses. The percentage of perseverations in block 2 (the percentage of anticipations to the location previously rewarded in block 1) per participant was calculated as an index of endogenous control of attention.

A modified version of the eye-tracking visual sequence learning task developed by Sheese et al. (2008) was administered to children at the age of 16 months. Stimuli consisted of a number of attractive cartoon pictures looming from 5 to 10 cm at a 150 ms rate accompanied by progressively decreasing tones (330 Hz, 392 Hz, and 262 Hz). Stimuli could appear in three different positions on the screen: left upper corner (position 1), right upper corner (position 2), and central bottom position (position 3) following a fixed sequence: 1, 2, 1, and 3. Each stimulus was presented for 3,500 ms. A 1-s blank screen (anticipatory period) appeared just before the next trial started. The complete task consisted of a total of eight complete sequences (32 trials). A total of 56 children performed this task. Children who attended less than 20 trials (*n* = 6) or had poor-quality data (*n* = 4) were excluded from the analyses. The proportion of corrected anticipated stimuli in the sequence for attended trials was calculated as a measure of endogenous control of attention.

##### 2.2.2.2. Behavioral measures

###### 2.2.2.2.1. Reverse categorization task

At the age of 2 years, children performed a version of the reverse categorization task by Carlson et al. (2004). Children were asked to classify 12 toy building blocks according to size (big blocks in a big box and small blocks in a small box). Before starting with the experimental procedure, the experimenter ensured that children could differentiate between the big and the small box and between big and small pieces. First, the experimenter classified the first six pieces while verbalizing the instructions. Then, children were encouraged to classify the remaining six pieces. Children had to correctly classify four pieces in a run to continue with the task. Next, children were asked to reverse the classifying rule: big blocks in the small box and small blocks in the big one. At the start of each trial, the experimenter gave children the corresponding instruction (e.g., “the small piece goes to the big box”), showing children the piece to be classified. To provide children with the pieces, the experimenter randomly took them from a tray with the constraint that no more than two pieces in a row were of the same size. A score was obtained by considering the number of correct trials after the change of rule. Toddlers who did not pass the pre-switch phase of the task were excluded from analyses (*n* = 5). We did not obtain data from two additional children who refused to collaborate on this task.

###### 2.2.2.2.2. Snack delay task

This task was also administered at the age of 2 years. Following the procedure designed by Kochanska et al. (2000), toddlers sat in front of a snack covered by a transparent plastic cup. The experimenter asked the children not to eat the snack until the experimenter rang a bell. Several runs with different waiting times (5, 10, 15, and 20 s) were conducted. Each trial started with the children placing his/her hands on a hands-shape mat 15 cm away from the snack. Children’s behavior during the waiting time was coded from 1 (ate the snack at the beginning of the trial) to 7 (waited for the entire trial). Children who waited without moving their hands from the mat were given 2 extra points. Children who did not show any preference for the snack were excluded from the analyses (*n* = 3). A final score was calculated by averaging the number of points that children got for each trial.

###### 2.2.2.2.3. Touch-screen spatial conflict task

A child-friendly version of the spatial conflict task with cartoons of animals as stimuli was performed by children at the age of 3 years (Gerardi-Caulton, 2000). Children were instructed to hold their hands on a hands-shaped mat. A looming black circle was displayed as a fixation point before each trial. Stimuli of the Spatial conflict task were presented using an ELO touch screen (Elo Touch Solutions, Inc.) connected to a computer running E-Prime 2.0. Children responded by touching right on the stimuli displayed on the screen. Monitor dimensions were 228.10 mm × 304.13 mm with a 1,024 × 768 pixels resolution and 60-Hz refresh rate. The experimenter initiated each trial by pressing a key once the children were attending to the center of the screen and their hands were on the mat. Two houses (7 cm), with a different animal inside each one, were displayed at both corners of the screen. One of these two animals (3 × 5 cm) also appeared at the same time above one of the houses, 2 cm away. The experimenter told the children to find the house of the animal above. Children were instructed to touch the left house with the left hand and the right house with the right hand. The experimenter encouraged the children to respond as fast as possible. The target was presented for 6 s, which was the time children had to respond. Trials with reaction times below 200 ms were not included in the analyses. When children made a correct response, cartoons were animated and accompanied by music. If children touched the wrong house or made no response, they heard a low beep sound, and the animals disappeared. The children performed three 8-trial blocks. A couple of animals appearing in each block were randomly selected from six different possible animals: frog, cat, pig, duck, monkey, and hedgehog. We calculated the conflict effect in reaction time by subtracting reaction times in the congruent condition from reaction times in the incongruent condition. Likewise, conflict effect accuracy was also calculated by subtracting the proportion of correct responses in the incongruent condition from the proportion of correct responses in the congruent condition. Smaller conflict effects reflect the better ability of IC.

###### 2.2.2.2.4. Delay of gratification task

At the age of 3 years, we used this task to observe children’s ability to delay immediate gratification (to eat a chocolate sweet or to get a colorful sticker) in order to get two times later (Lemmon and Moore, 2007). First, we asked the children to point to their favorite prize (the chocolate sweet or the sticker) to start the task with their preferred option. In each trial, the experimenter asked children to choose between having one chocolate sweet/sticker immediately and keeping two in a bag that they would have at the end of the experimental session. Children completed a total of 12 trials (six with the chocolate sweets and six with the stickers). The number of choices to delay was used as a measure.

###### 2.2.2.2.5. Electroencephalography measures

This technique was used at 5 years of age. We registered EEG signal while children performed a Go/No-go task. Children were seated in front of a screen at 60 cm. Stimuli were displayed on 1,024 × 768 pixels, 19-inch monitor (60 Hz). A high-density EGI system (EGI’s Geodesic Sensor Net, Eugene, Oregon) was used to obtain the electrical brain activity of children. Stimuli were presented in a Windows XP PC with E-Prime 2.0.8 synchronized with the EGI system. We used pediatric 128 sensor nets with Ag/AgCl electrodes embedded in small soft sponges soaked in an electrolyte solution. Impedances were kept below 50 KΩ before acquisition. The EEG signal was registered at a sampling rate of 250 Hz, and the acquisition was filtered employing elliptical low-pass (100 Hz) and high-pass (0.1 Hz) hardware filters, being online referenced to the vertex channel. A 50-Hz notch filter was additionally applied during the signal recording FIR filter (roll-off = 2 Hz, stopband gain-53 dB, passband gain = −0.1 dB).

**Figure 1:**
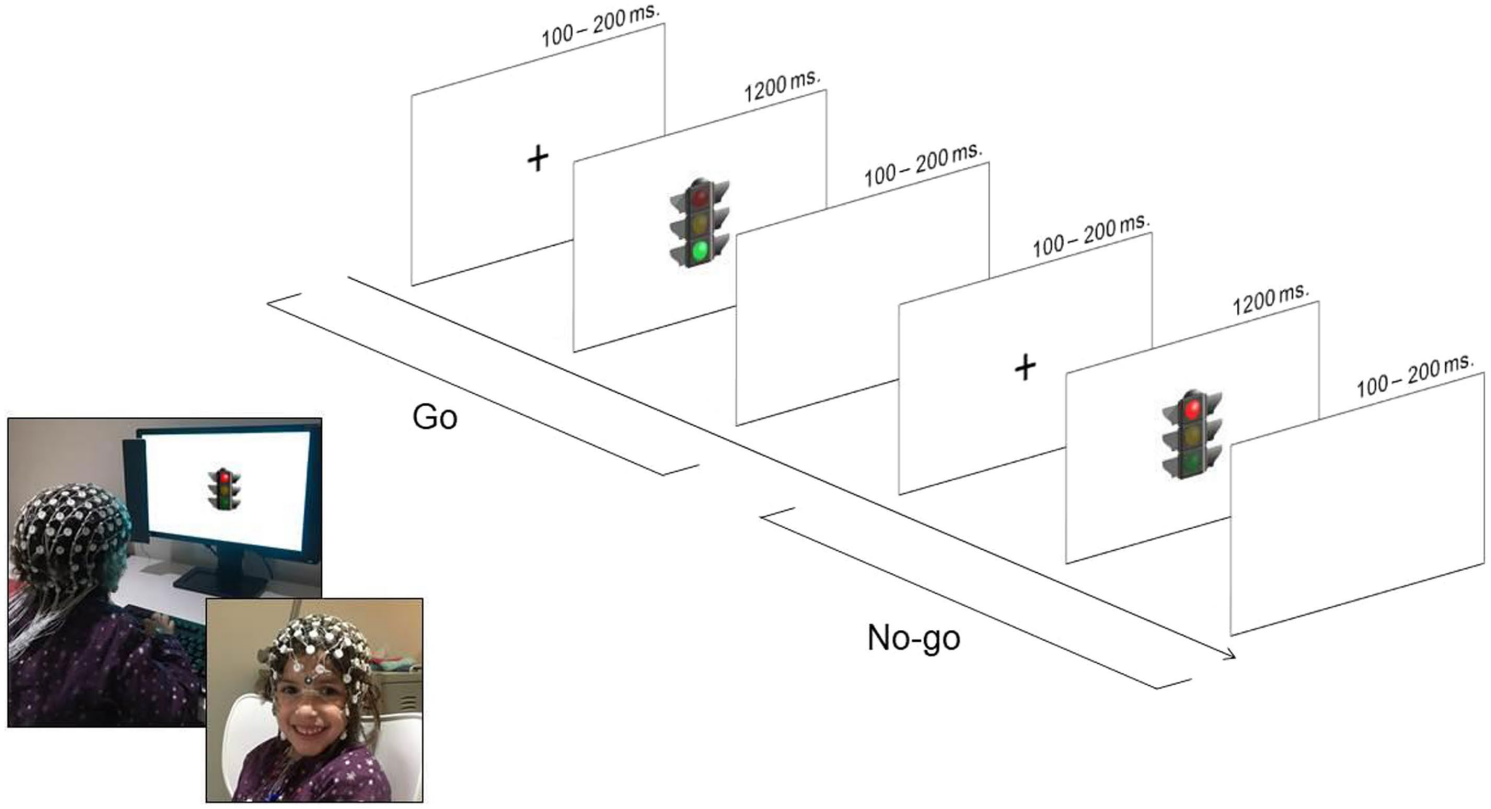
Go/No-go task procedure.

We used a child-friendly version of the Go/No-go while EEG was recording. Children were instructed to press a button as fast as possible in a response box every time a traffic light showed the green color (Go trials), withholding the response when the traffic light displayed the red color (No-go trials). We asked children to put their fingers on the button during the entire duration of the task in order to be prepared to respond. In order to motivate children to do their best, we contextualized the task as a car race game. It consisted of two blocks of 60 trials: 40 Go trials and 20 No-go trials. Experimental trials started with a fixation point of random duration between 150 and 300 ms followed by target stimuli (traffic light), which were presented until children gave a response with a maximum duration of 1,200 ms. Trials with responses faster than 200 ms were excluded from analyses. A blank screen after target stimuli was set at random between 100 and 200 ms. Children were given general feedback about their performance at the end of each block. The experimental task started after a practice block (12 trials) in which children received auditory feedback (a tone) indicating whether they gave a correct or incorrect response in the trial to ensure children understood the task. This auditory feedback was absent in experimental trials. The practice block could be repeated if children showed difficulties with the task instructions. Stimuli and the schema for the task procedure are illustrated in Figure 1. The proportion of errors in No-go trials and the proportion of omissions in the Go trials and d’ were calculated as behavioral measures in this task following prior research (Miller, 1996).

Maryland Analyses of Developmental EEG (MADE) pipeline scripts (Debnath et al., 2020) were used for the pre-processing of EEG data in EEGlab (Delorme and Makeig, 2004). In brief, the pipeline removes bad channels employing the FASTER plugin (Nolan et al., 2010) and filtered signal by applying a high-pass and low-pass filter (0.2–30 Hz). In order to improve ICA performance, we created a copy of the data set, high-pass filtered the data (1 Hz), and segmented it in 1 second epochs. Epochs containing excessive EMG or unusually high or low amplitudes (±1,000 μV) were removed. After that, we conducted ICA in the copy data set and transferred the ICA weights to the original data set, where eye-blink and eye-movement components were removed employing the adjusted-ADJUST EEGlab plugin (Leach et al., 2020). Subsequently, data were epoched in 1000 ms segments (200 ms baseline) locked to the presentation of the stimuli. Then, automatic artifact rejection was performed by interpolating those channels (±125 μV). In the event that the percentage of channels interpolated of the epochs exceeded 20%, the segment was removed. Initial channels removed by the FASTER plugin were spherically interpolated, and we re-referenced to the average. Finally, we visually inspected the data in order to remove bad epochs after the automatic cleaning. Children should have at least 12 trials per condition with clean EEG data to be included in ERPs analyses. Peak amplitude and latency of N2 were explored in a time window from 300 to 400 ms after stimulus onset in a selection of frontocentral leads around FCz (corresponding to channels number 107, 7, 6, 13, and 113 according to the EGI system), whereas peak amplitude and latency of P3 have explored between 400 and 800 ms in electrodes surrounding Pz (61, 68, 79 channels). Peak latency per condition and differences in peak amplitude between conditions were calculated as neural markers of IC for each component. To calculate the difference peak amplitude of the N2 component, we subtracted the Go minus No-go condition, whereas the Go condition was subtracted from the No-go condition for the P3 component. Thus, greater positive values in both cases indicated a larger magnitude of the difference. More specifically, larger values indicate increased negativity of the N2 while increased positivity of the P3 for the No-go condition relative to the Go condition.

#### 2.2.3. Analyses plan

Analyses were performed using IBM SPSS Statistics 25 software and RStudio 2022.02.3 with *lavaan* package (Rosseel, 2012) for structural equation modeling (SEM). Pairwise correlations analyses were conducted to explore the relationship between IC-related skills measured over time in the first years of life (9 months to 3 years of age) and test the relationship between early measures of IC-related skills and performance of children in an IC task at the behavioral and neural level at 5 years of age (outcome measures). Pearson correlation coefficients were calculated. In addition, we computed the 95% confidence intervals (CIs) for the correlation coefficients to check the precision to which they were estimated. The *!rhoCImacro* for SPSS (Weaver and Koopman, 2014) was used to estimate bootstrapped 95% CIs taking 1,000 samples. The width of the CI would also inform about the accuracy to which correlation parameters are estimated, with narrower intervals indicating a more precise estimation (McDonald, 2014). Moreover, it has been proposed that CIs may additionally help to decide about rejecting or not the null hypothesis. Whenever the upper or lower bounds of CIs cross, the 0 value is generally interpreted as indicating that null hypotheses cannot be completely discarded (Cumming, 2014). Differences between Go and No-go conditions in the Go/No-go task with regard to the ERPs were checked with *T*-tests. Structural Equation Modeling was applied to test the specific contribution of EA and IC skills over time to predict individual differences in IC at 5 years of age. The SEM intends to test the causal relations among measures throughout a regression system based on a set of related theoretical hypotheses (Ullman and Bentler, 2012). A path model was built in which EA and basic IC skills measures (observed variables) were set as predictors of behavioral IC (latent factor) and neural markers of IC (observed variables) at the age of 5 years. Only the most relevant variables were included in the model for parsimony. This decision was guided by both theory (consistency among measures) and exploratory correlation analyses. As Little’s MCAR test for missing data revealed that data were completely missing at random (*p* > 0.05), the full-information maximum likelihood was used to handle missing data across tasks (McNeish, 2017).

## 3. Results

### 3.1. Descriptive analyses

Table 1 summarizes the descriptive statistics for all measures from the different tasks administered at each wave. Mean, range, and standard deviation are provided.

### 3.2. Relationship between infancy and toddlerhood measures of executive attention and inhibitory control

As shown in Table 2, a higher proportion of perseverations in the Shifting task at 9 months was associated with more perseverative errors in the Reverse Categorization task at 2 years of age (*r* = −0.42, *p* = 0.02, 95% CI [−0.71, −0.03]). The ability of children to delay at 2 and 3 years of age was also positively related (*r* = 0.34, *p* = 0.02, 95% CI [0.07, 0.56]). Anticipations in the Visual Sequence Learning task at 16 months were not correlated to any of the measures at any of the time points within this range.

**Table 2:** Correlations among different measures of executive attention in infancy and toddlerhood.

		1.	2.	3.	4.	5.	6.
9 months	1. Shifting task (% Perseverations)	–	–	–	–	–	–
16 months	2. VSL task (% correct anticipations)	0.24 [−0.23, 0.63]	–	–	–	–	–
2 years	3. Reverse categorization task (*z* score)	−0.42* [−0.71, −0.03]	0.19 [−0.35, 0.58]	–	–	–	–
	4. Snack Delay task (*z* score)	−0.25 [−0.53, 0.19]	0.04 [−0.28, 0.31]	0.29* [0.00, 0.69]	–	–	–
3 years	5. Spatial conflict task (Conflict score, RT)	0.09 [−0.11, 0.28]	−0.26 [−0.18, 0.59]	0.11 [−0.27, 46]	−0.01 [−0.33, 0.48]	–	–
	6. Spatial conflict task (Conflict score, ACC)	−0.00 [−0.36, 0.33]	−0.09 [−0.48, 0.23]	0.02 [−0.36, 0.39]	−0.22 [−0.43, 0.23]	−0.14 [−0.40, 0.14]	–
	7. Delay of gratification task (*z* score)	−0.06 [−0.45, 0.35]	−0.15 [−0.51, 0.26]	−0.00 [−0.36, 0.36]	0.34* [0.07, 0.56]	−0.05 [−0.32, 0.23]	0.06 [−0.33, 0.48]

*p < 0.05; 95% confidence intervals after bootstrapping with 1,000 samples in square brackets. Significant correlations according to both *p*-value and CI interpretation are highlighted in bold.

### 3.3. Statistical analyses exploring ERPs

**Figure 2:**
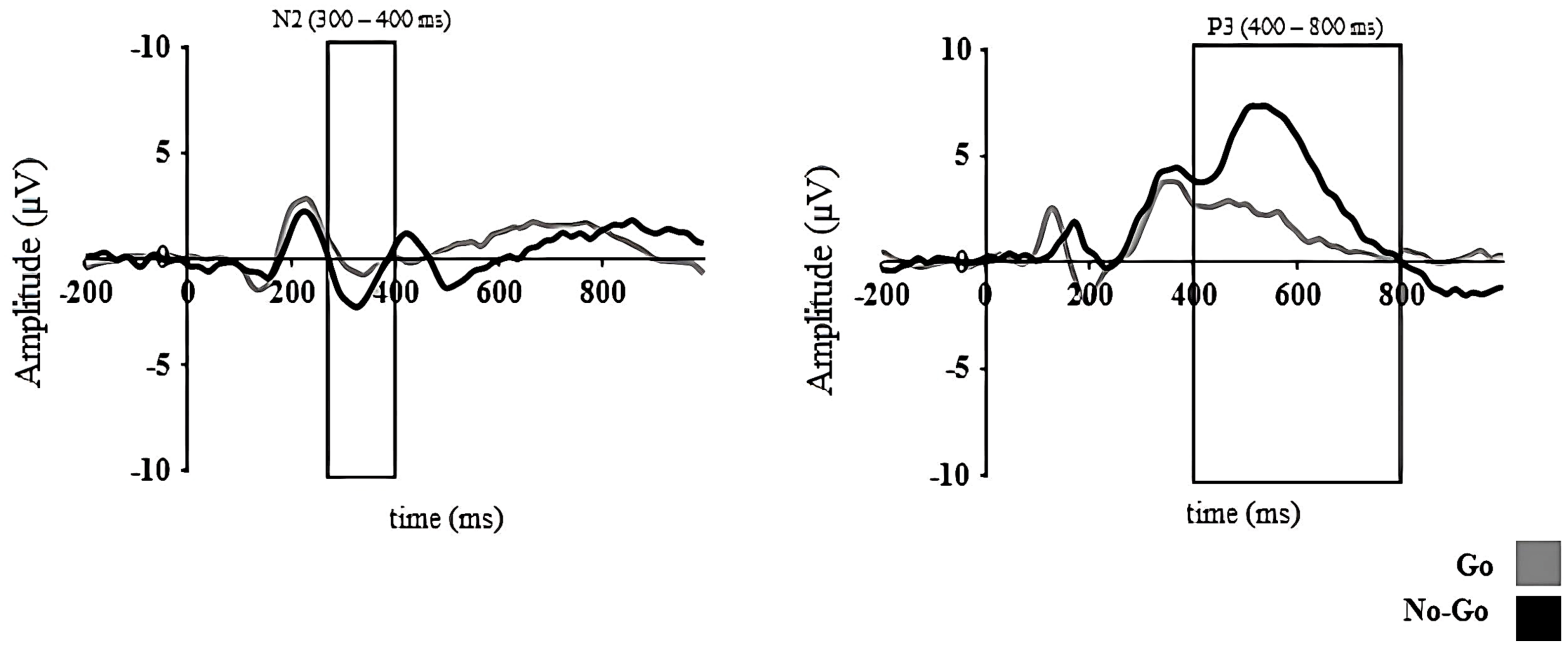
N2 and P3 ERP components linked to the target processing in the Go/No-go task. Average ERPs of a set of electrodes around FCz and Pz are shown for N2 and P3 components, respectively.

**Figure 3:**
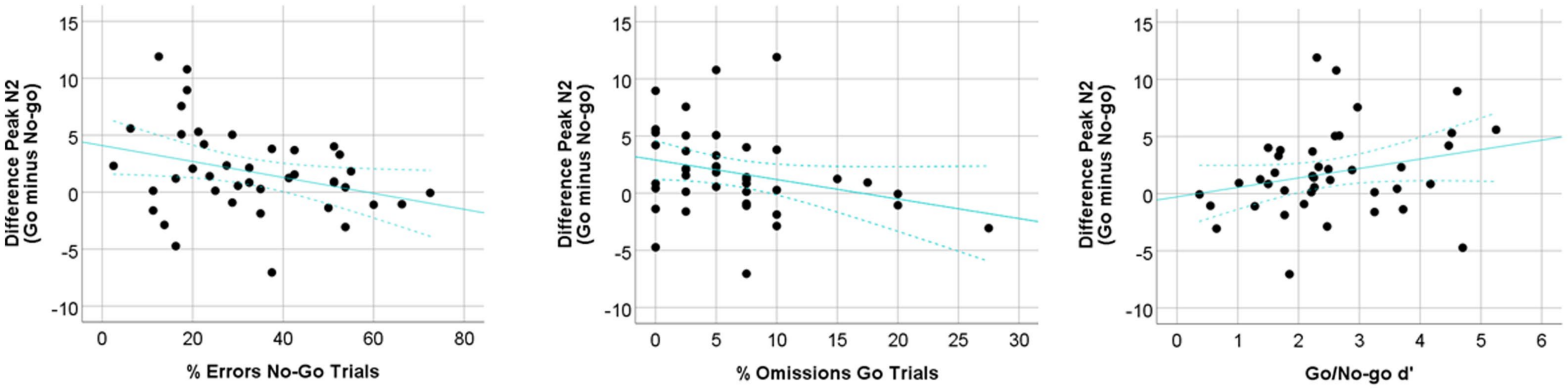
Correlation plots for the Go/No-go behavioral measures and the difference in peak amplitude (Go minus No-go condition) of the N2 ERP component.

*T*-tests revealed significantly larger peak amplitudes of N2 and P3 in the No-go vs. the Go condition (*t*_42_ = 2.49, *p* = 0.02 and *t*_42_ = −5.01, *p* < 0.001, respectively; see Figure 2). No differences among conditions were observed in peak latency for either N2 (*t*_42_ = 0.86, *p* = 0.39) or P3 component (*t*_42_ = 0.02, *p* = 0.98). Correlation analyses (see Table 3) showed that the N2 component was associated with children’s performance in the Go/No-go task. As seen in Figure 3, the smaller peak amplitude difference in the N2 component (which reflects a smaller negative deflection of the N2 component for the No-go trials relative to the Go trials), the greater commission errors in the No-go trials (*r* = −0.33, *p* = 0.02, 95% CI [−0.52, −0.10]) and omission errors (*r* = −0.26, *p* = 0.05, 95% CI [−0.48, −0.04]). The difference in peak amplitude of the N2 also showed a significant positive correlation with d’ (*r* = 0.28, *p* = 0.04, 95% CI [0.01, 0.53]). However, the peak amplitude difference for the P3 component was unrelated to behavioral measures of the Go/No-go task. There was also a lack of association among latency of N2 or P3 components with behavioral measures in the Go/No-go task. Association between measures at infancy and toddlerhood and IC indicators at 5 years of age in the Go/No-go task.

**Table 3:** Correlations between behavior indicators and neural measures in the Go/No-go at 5  years of age.

		% omissions Go	% errors No-go	d’
Amplitude	Difference Peak N2	−0.26* [−0.51, −0.03]	−0.33* [−0.57, −0.07]	0.28* [0.01, 0.53]
	Difference Peak P3	−0.11 [−0.29, 0.06]	−0.05 [−0.36, 0.22]	0.11 [−0.10, 0.33]
Latency	N2 Go	−0.10 [−0.31, 0.14]	–	0.21 [−0.06, 0.43]
	N2 No-go	–	0.00 [−0.33, 0.33]	−0.14 [−0.49, 0.20]
	P3 Go	0.25 [−0.21, 0.61]	–	−0.23 [−0.58, 0.20]
	P3 No-go	–	0.29* [−0.04, 0.56]	−0.22 [−0.57, 0.25]

*p < 0.05; 95% confidence intervals after bootstrapping with 1,000 samples in square brackets. Significant correlations according to both *p*-value and CI interpretation are highlighted in bold. N2 difference peak amplitude = Go minus No-go condition. P3 difference peak amplitude = No-go minus Go condition. Only ERP latencies and behavioral measures for the same experimental condition were correlated.

Correlation analyses are summarized in Table 4. The performance in the Reverse Categorization task at the age of 2 years was negatively related to the proportion of errors in No-go trials (*r* = −0.43, *p* = 0.02, 95% CI [−0.73, −0.12]). It showed increased sensitivity (d’) in the Go/No-go task (*r* = 0.37, *p* = 0.04, 95% CI [0.04, 0.61]). None of the IC measures at the age of 3 years were associated with IC at the age of 5.

**Table 4:** Correlations between early measures of executive attention and inhibitory control indicators at 5  years of age.

		% omissions Go	% errors No-go	d’	Difference Peak N2	Difference Peak P3
9 months	Shifting task (% Perseverations)	0.01 [−0.33, 0.46]	−0.00 [−0.44, 0.44]	−0.12 [−0.51, 0.25]	−0.01 [−0.11, 0.27]	0.20 [−0.20, 0.55]
16 months	VSL task (% correct anticipations)	−0.36* [−0.63, 0.07]	−0.06 [−0.43, 0.37]	0.11 [−0.34, 48]	0.13 [−0.29, 0.52]	0.22 [−0.18, 0.58]
2 years	Reverse categorization task (*z* score)	−0.37* [−0.66, 0.01]	−0.43* [−0.73, −0.12]	0.37* [0.04, 0.61]	−0.10 [−0.27, 0.52]	−0.09 [−0.47, 0.53]
	Snack Delay task (*z* score)	−0.33* [−0.65, 0.08]	−0.13 [−0.51, 0.29]	0.20 [−0.22, 0.56]	0.25 [−0.51, 0.00]	0.16 [−0.10, 0.41]
3 years	Spatial conflict task (Conflict score, RT)	0.03 [−0.21, 0.28]	−0.21 [−0.46, 0.16]	0.14 [−0.15, 0.36]	0.05 [−0.35, 0.30]	0.10 [−0.36, 0.24]
	Spatial conflict task (Conflict score, ACC)	0.05 [−0.27, 0.38]	−0.05 [−0.40, 0.33]	−0.09 [−0.45, 0.33]	−0.11 [−0.39, 0.13]	−0.14 [−0.36, 0.08]
	Delay of gratification task (*z* score)	0.08 [−0.25, 0.49]	−0.10 [−0.41, 0.23]	0.02 [−0.34, 0.30]	0.08 [−0.23, 0.38]	−0.08 [−0.41, 0.27]

*p < 0.05; 95% confidence intervals after bootstrapping with 1,000 samples in square brackets. Significant correlations according to both *p*-value and CI interpretation are highlighted in bold.

### 3.4. Structural equation model for predicting inhibitory control at 5  years of age

**Figure 4:**
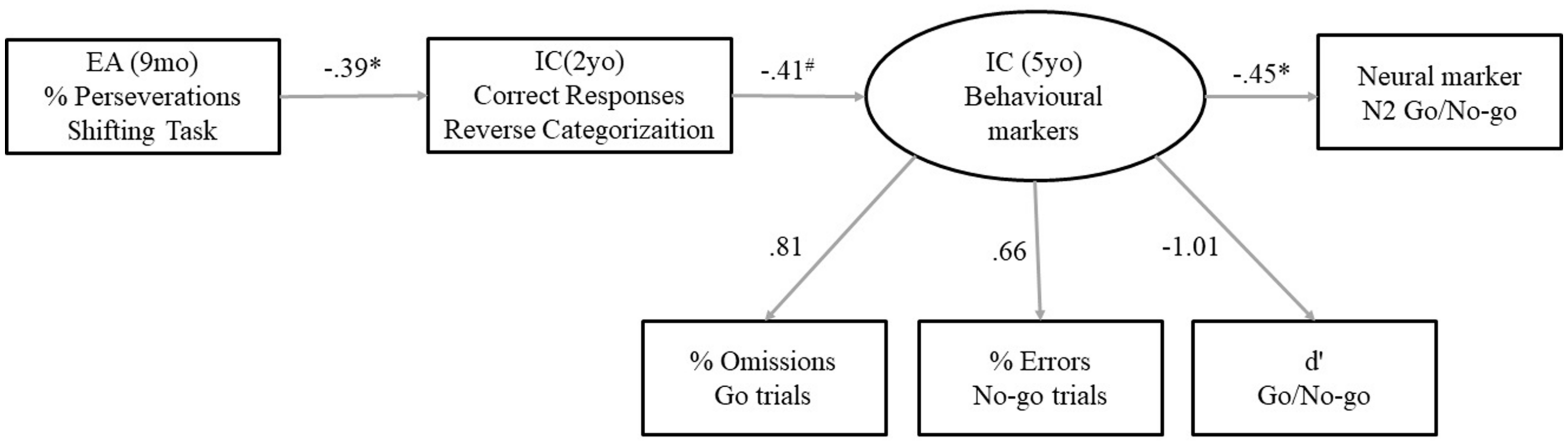
Structural equations model to predict IC at 5  years of age from EA in infancy and IC in toddlerhood.

A model to predict IC from EA at 9 months of age (using the performance of children in the shifting task) and IC at 2 years of age (with performance in the Reverse Categorization task as an indicator) was built. A latent variable from commission errors, omissions, and d’ in the Go/No-go task was created as a general outcome measure of the IC at the behavioral level at the age of 5 years. The No-go vs. Go difference in peak amplitude of the N2 was also introduced in the model as a neural marker of IC at 5 years of age and predicted from behavioral IC at the same age. The overall model adjusted to the data (*χ*^2^(9) = 12.11, *p* = 0.21) with an acceptable fit (RMSA = 0.08). As shown in Figure 4, a lower proportion of perseverations in the shifting task at 9 months of age predicted the performance of children in the Reverse Categorization at 2 years. At the same time, performance in the Go/No-go was predicted by IC at 2 years of age, although the estimate for this prediction was only marginally significant (*p* = 0.06). Finally, the Go vs. No-go N2 peak difference as a neural marker of IC was successfully predicted from the composite of the behavioral indicators in the Go/No-go.

## 4. Discussion

The main goal of the present research was to investigate, from a longitudinal perspective, the development of individual differences in IC at the age of 5 years from infancy and toddlerhood. The literature on IC development has typically focused on the study of IC from the preschool years onward, and little is still known about the development of IC during the first years of life. This study intended to address the existing gap in developmental research by exploring early EA and IC skills accounting for these individual differences in IC at 5 years of age. For that purpose, we conducted a five-wave longitudinal study in which EA was assessed at 9 and 16 months and IC at 2, 3, and 5 years of age. Overall, our results revealed that EA and basic IC skills could be assessed as early as infancy and toddlerhood to predict IC later in early childhood. We found that EA at 9 and 16 months of age was associated with individual differences in IC at 2 and 5 years of age, following distinct patterns of association with IC at each age depending on the EA skill measured. In our predictive model, EA at 9 months of age was a predictor of IC at 2 years of age, with IC predicting IC at 5 years in a successive way. Our results give further support to a hierarchical model of IC development (Garon et al., 2008; Hendry et al., 2016).

We observed that IC skills at 2 years were the most consistent predictors of IC at the age of 5. In particular, the performance of children at the age of 2 in the Reverse Categorization task (a task measuring set shifting) was related to two of the behavioral indexes provided by the Go/No-go task: the percentage of commission errors in No-go trials (that represents a failure in inhibiting the tendency to respond) and d’ (which indicates the specificity of their inhibition behavior in relation to the experimental condition). However, the self-control that 2-year-olds demonstrated in the Snack Delay task was not related to their performance in the Go/No-go task at 5 years of age. These results partially confirmed our hypotheses about the relationship between IC skills in toddlerhood and early childhood. However, we expected that both IC, as measured with the Reverse Categorization task and the Snack Delay task, would be related equally to performance in the Go/No-go. One possible reason for this different pattern of association is the fact that the Reverse Categorization and the Snack Delay tasks are tapping different aspects of IC. The distinction between “cold” and “hot” IC skills is highly accepted. Whereas “hot” IC refers to the ability to self-control in arousing situations (such as resisting the temptation of eating a treat), “cold” IC refers to cognitive control in non-emotional situations (Welsh et al., 2014). Factorial analyses of IC measures have frequently found two factors corresponding to these two facets of IC (Hongwanishkul et al., 2005; Zelazo and Carlson, 2012). Our results may indicate that the development of “cold” and “hot” IC skills diverges from toddlerhood. The fact that the performance in the Go/No-go task at 5 years was only clearly associated with the performance in the Reverse Categorization task at 2 years of age could be attributed to the fact that both can be considered as measuring “cold” IC. Further research should independently explore the developmental trajectories of the two factors in order to confirm this idea.

In general, the tasks in our study that seem to theoretically target the same core IC functions were indeed more prone to be associated over time. This applies to, for example, the Shifting and Reverse Categorization tasks administered at 9 months and 2 years of age, respectively. Both tasks require changing the mindset from the first to the second block of trials having the same structure, presumably tapping the same IC component (Carlson, 2005; Diamond, 2013; Conejero and Rueda, 2017). The only difference between the two tasks is the response modality. While the Shifting task depends on the infant’s looking behavior, the Reverse Categorization task relies on children’s motor behavior. In the same line, IC in the delay tasks at 2 and 3 years of age is also intercorrelated; they are foreseeably intended to measure the same aspect of IC (i.e., self-control), which would explain the shared variance over time (Kochanska et al., 2000; Mulder et al., 2014). By contrast, the two measures of EA collected at 9 (Shifting task) and 16 months of age (Visual Sequence Learning task) were not associated. It may well be the case that different core EA mechanisms are underlying EA tasks at 9 and 16 months of age. In addition, performance in the Visual Sequence Learning task at 16 months was unrelated to any IC measure at any age, except for a trend to associate with omissions in the Go/No-go task at 5 years of age (i.e., the fewer anticipations in the Visual Sequence task, the greater proportion of omitted trials in the Go condition). In this respect, omissions in the Go/No-go task have been interpreted as a consequence of a failure in sustained attention (Lewis et al., 2017b). Similarly, learning to anticipate in the Visual Sequence Learning task also requires maintaining attention to the stimuli presented to create a representation of the sequence (Rothbart et al., 2003). Therefore, the ability to sustain attention might have more weight to explain individual differences in the proportion of anticipations in the Visual Sequence Learning task at 16 months of age. By contrast, the Shifting task could capture individual differences in attention flexibility, a cognitive ability more closely linked to IC. However, this tentative explanation should be further explored.

In line with prior longitudinal research on the early development of IC, continuity across ages in the relation among the different IC measures was not always found (Holmboe et al., 2008; Joyce et al., 2016; Kloo and Sodian, 2017). None of the IC measures at 3 years of age were related to IC at 5 years, and only the delay tasks were correlated between the 2nd and 3rd year of life. While these inconsistencies in the results may be thought to be driven by differences in the characteristics of stimuli and procedure [e.g., computerized vs. manipulative tasks (Leeson, 2006)], this appears to be unlikely in our case given that some tasks were not associated over time even when the methodology did not vary over time. This is the case of the 9-and 16-month-old measures of EA, which were unrelated despite both being computerized eye-tracking measures with a similar kind of stimuli. Even more, the different modalities of the tasks did not compromise the relationship among the measures at 9 months and 2 years of age. The suggestion has been recently raised that IC development follows a heterotypic continuity (Petersen et al., 2016). In this connection, measuring longitudinal changes in IC should account for the significant aspects underpinning IC at each developmental moment requiring the selection of the most appropriate measures of IC at each point. For instance, to return to our study, the delay task at 2 years of age might be working better in distinguishing individual differences in IC than the delay task at 3 years (waiting for an increasing time interval might be a more sensitive measure of self-control). Consequently, the sensitivity of the experimental tasks at each age should be taken into account when designing longitudinal studies investigating IC. In doing so, it is possible to broaden the age range in which the longitudinal development of IC can be investigated while capturing individual differences in IC more effectively. Along with this, ensuring the overlapping of some measures between contiguous ages (Petersen et al., 2020) may help to overcome the problem of unexplained discontinuities in development that are often found in longitudinal research.

About the ERPs in the Go/No-go task at 5 years of age, in consonance with the literature, the N2 and P3 were more pronounced in the No-go than in the Go condition (Lahat, 2010; Abdul Rahman et al., 2017; Sullivan et al., 2022). Unlike other studies (Sullivan et al., 2022), no differences in peak latency were found for N2 or P3 components. Only the difference peak amplitude in the N2 component was concurrently correlated with behavioral indicators of IC. Larger N2 peak differences were observed for children who committed fewer No-go errors and had better discriminability between Go and No-go targets. This is in agreement with evidence supporting that the N2 reflects neural processes of task monitoring and inhibition (Lo, 2018). In contrast to some prior studies (Willner et al., 2015; Abdul Rahman et al., 2017; Huster et al., 2020), we failed to find a significant relationship between P3 and performance in the Go/No-go. Nevertheless, the correlation of N2 and P3 amplitudes with behavioral measures in the Go/No-go task is not always found in the literature regarding the pediatric population (Jonkman et al., 2003). For example, a recent study found that N2 difference peak amplitude was uncorrelated to performance in 5-year-olds (Sullivan et al., 2022). This discrepancy in the results could be explained by the fact that in this study, a general accuracy measure was used instead of focusing on more specific measures of IC (such as accuracy in No-go trials or d’). Furthermore, it has been proposed that N2 and P3 are underlying different IC processes (Ciesielski et al., 2004; Jonkman, 2006; Huster et al., 2013): The earlier N2 component is thought to be involved in the detection of a situation in which IC must be applied (i.e., when some information or different response options are in conflict), whereas the P3 component in the implementation of the inhibition having a more protracted development (Jonkman, 2006). None of the measures of EA and IC at younger ages were directly associated with functional brain activity linked to IC. However, EA at 9 months and IC at 2 years of age were indirectly associated through its association with the overall performance of children in the Go/No-go, which predicted an increased difference in the N2 component between the Go and No-go trials. It has been proposed that the increment in the amplitude of N2 in early development indicates the maturation of neural processes underpinning the improvements in IC (Buss et al., 2011; Abundis-Gutiérrez et al., 2014). In consonance with this, our model suggests that infants showing better EA capacity will show enhanced IC skills in toddlerhood, leading to more efficient IC and greater maturation of IC brain mechanisms as they grow into preschoolers.

To sum up, our results point to the possibility of predicting children’s IC from infancy and toddlerhood. Our data show a consistent relationship between some of the mechanisms that undertake the inhibition of response over time, corroborating the idea that EA and IC measures in infancy and toddlerhood capture a basic IC mechanism that develops into more complex forms of IC later on (Garon et al., 2008; Casey et al., 2011; Hendry et al., 2016). In addition, our data give further support to the idea that IC can be traced back to infancy and toddlerhood, extending evidence from prior longitudinal studies encompassing from 9 to 24 months of age (Holmboe et al., 2008; Johansson et al., 2015; Miller and Marcovitch, 2015) or toddlerhood (Hughes and Ensor, 2011; Gagne and Saudino, 2016; Kloo and Sodian, 2017; Veer et al., 2017). One of the main strengths of this study is the longitudinal design. Unlike cross-sectional studies, longitudinal studies become indispensable when trying to trace the individual pathways of IC to predict different developmental outcomes (Karmiloff-Smith et al., 2018). However, existing longitudinal research on the emergence of IC skills has mainly concerned short developmental spans (from months to a couple of years). To our knowledge, there are no longitudinal studies on IC development comprising the full period from infancy to 5 years of age in the current literature. As far as we know, no prior studies have found a longitudinal relationship between infants’ EA skills and IC beyond the age of 2 years. Data from the current study stress the relevance of the first 5 years of life for the development of IC, pointing to the 9th month of life as a key starting point to explore the longitudinal development of IC coinciding with the remarkable change in the endogenous control of attention occurring from this age (Kannass et al., 2006). Further longitudinal research extending the developmental period is needed in order to understand how the transition from basic forms to more complex mechanisms of IC (i.e., inhibitory processes in reaction time in Go/No-go tasks) takes place. Addressing this question longitudinally sheds light on the origins of individual differences during infancy that is still observed later in childhood.

The main limitation of this study is the relatively small sample size. This made it not advisable to test complex models predicting IC through SEM as these analyses may lead to biased results (McNeish, 2017). Likewise, our sample is possibly not big enough to find statistically significant correlations or to correct for the number of comparisons. We cannot completely discard that some non-significant results in correlation analyses could be due to a lack of statistical power to detect feasible relations between variables. For significant correlations, *p*-values are not smaller than 0.02. Thus, multiple comparisons correction might not be appropriate to control for type I error in the current study without assuming a high risk of type II error (Tyler, 2018; White et al., 2019) as we may conclude that none of the correlations are statistically significant, neglecting any true association among variables. For instance, using the Bonferroni method, the critical *p*-value for 21 tests decreases from 0.05 to 0.002, whereas the less conservative Benjamin–Hochberg method gives critical *p*-values from 0.002 to 0.02 for the first 10 correlations in the rank (McDonald, 2014). For this reason, some criticisms have emerged in recent years about lowering alpha values methods to control for false positives (Lakens et al., 2018). Alternatively, the estimated CIs for the correlation coefficients may serve to judge the robustness of the estimates and the replicability of the effects. It is noteworthy that we generally obtained wide CIs, which may indicate that coefficients were poorly estimated. This is a common problem in psychological research, as CIs only narrow down with very large sample sizes (Brand and Bradley, 2016). Overall, it would be desirable to increase the sample size in future studies to test the replication of the current findings, which would also allow the building of more complex predictive models.

Furthermore, the use of various tasks assessing different aspects of EA and IC at each age caused heterogeneity of the measures, which were not always equivalent across ages. This is a common limitation in longitudinal studies addressing early cognitive development (Hendry et al., 2016; Conejero and Rueda, 2017) as most of these studies have to rely on different tasks to be adjusted to children’s age. This impairs testing the longitudinal growth of IC. Except for some tasks, such as the one being developed by Hendry et al. (2021), it is difficult to find just a single task to evaluate IC from infancy to early childhood. Notwithstanding this limitation, using age-appropriate measures each time has the advantage of representatively measuring individual differences of IC at different ages. It has been proposed that IC might qualitatively differ from age to age (Petersen et al., 2016). In addition, this approach allows for investigating the foundations of IC according to the hierarchical model of IC development (Garon et al., 2008; Hendry et al., 2016). Much research is still needed to analyze the sensitivity and validity of IC measures at every age, following Carlson (2005) and Garon et al. (2014) in a more recent attempt to test the properties of some existing IC measures. In addition, increasing the consistency among measures over time might increment the predictive value. Incorporating EEG measures over time will also improve the understanding of the joint development of the behavioral changes of IC in relation to the underlying neural mechanisms. The use of new EEG analysis approaches beyond ERPs analyses may also help to expand our understanding of the developmental processes underlying the development of IC processes. For example, the analyses of functional brain connectivity may shed light on the development of neural networks parallel to the enhancement of IC skills (Park and Friston, 1979; Shovon et al., 2017), whereas the multivariate pattern analyses could provide richer information about brain processing by identifying neural patterns of activation related to IC (Ashton et al., 2022).

Finally, this research has some practical implications. It offers insights into how EA processes and elementary forms of IC separately contribute to the typical development of IC in the first 5 years of life. This knowledge may help to target intervention by focusing on the most determinant aspects fostering IC development at each age. Furthermore, results from this study draw attention to the importance of promoting IC as early as infancy. Evidence suggests that the earlier the intervention, the greater the impact (Wass et al., 2012). Finally, further research is also needed to explore early antecedents of IC, such as EA, and developmental trajectories of IC in relation to different outcomes in childhood and adolescence for typical and atypical development. In the future, this may lead to anticipating earlier possible IC-related problems, which is the first step for prevention. This is especially relevant for children at risk for academic underperformance (Lengua, 2003; Clark et al., 2010; Rueda et al., 2010; Blair, 2016), developmental disorders such as Attention-Deficit/Hyperactivity Disorder (Adams et al., 2008), or premature children (Baron et al., 2012; Réveillon et al., 2016), all circumstances associated with poorer IC functioning. Research exploring different factors influencing the longitudinal development of IC will also provide new insights for predicting IC-related developmental outcomes.

## Data availability statement

The raw data supporting the conclusions of this article will be made available by the authors, without undue reservation.

## Ethics statement

The studies involving human participants were reviewed and approved by University of Granada ethics committee. Written informed consent to participate in this study was provided by the participants’ legal guardian/next of kin. Written informed consent was obtained from the individual(s) for the publication of any identifiable images or data included in this article.

## Author contributions

ÁC was involved in conceptualization, study design, recruitment, task programming, data acquisition, data processing, statistical analyses, and writing the manuscript. JR-P was dedicated to the pre-processing and analyses of EEG or ERP data. SM took part in the pre-processing of part of the behavioral data. ÁH collaborated in the pre-processing of EEG or ERP data. MR was the main supervisor of this project and participated in the conceptualization, study design, and review of the manuscript draft. All authors contributed to the article and approved the submitted version.

## Funding

This research was supported by the Spanish State Research Agency (grants PSI2017-82670-P and PID2020-113996GB-100), awarded to MR, and a FPU fellowship (AP2010-3525) awarded to the main author ÁC from the Spanish Government.

## Conflict of interest

The authors declare that the research was conducted in the absence of any commercial or financial relationships that could be construed as a potential conflict of interest.

## Publisher’s note

All claims expressed in this article are solely those of the authors and do not necessarily represent those of their affiliated organizations, or those of the publisher, the editors and the reviewers. Any product that may be evaluated in this article, or claim that may be made by its manufacturer, is not guaranteed or endorsed by the publisher.
